# Discrimination between Pancreatic Cancer, Pancreatitis and Healthy Controls Using Urinary Polyamine Panel

**DOI:** 10.1177/10732748211039762

**Published:** 2022-02-08

**Authors:** Samuli I. Nissinen, Markus Venäläinen, Pekka Kumpulainen, Antti Roine, Merja R. Häkkinen, Jouko Vepsäläinen, Niku Oksala, Tuomo Rantanen

**Affiliations:** 1 Department of Internal Medicine, School of Medicine, 205537 University of Eastern Finland, Kuopio, Finland; 2 Department of Internal Medicine, 3701 Kanta-Häme Central Hospital, Hämeenlinna, Finland; 3 60670 Tampere University Hospital, Tampere, Finland; 4 Faculty of Medicine and Health Technology, 7840 Tampere University, Tampere, Finland; 5 School of Pharmacy, Biocenter Kuopio, 205537 University of Eastern Finland, Kuopio, Finland; 6 Centre for Vascular Surgery and Interventional Radiology, 60670 Tampere University Hospital, Tampere, Finland; 7 Department of Surgery, School of Medicine, 205537 University of Eastern Finland, Kuopio, Finland

**Keywords:** pancreatic cancer, urine VOCs, polyamines, quadratic discriminant analysis, biomarker

## Abstract

**Backround:**

Polyamines play an important role in cellular proliferation, and the change in polyamine metabolism is reported in various cancers. We searched for urinary polyamine signature for distinguishing between pancreatic cancer, premalignant lesions of the pancreas (PLP), acute and chronic pancreatitis, and controls.

**Methods:**

Patients and controls were prospectively recruited in three Finnish hospitals between October 2013 and June 2016. The patients provided a urine sample at the time of the diagnosis. The panel of 14 polyamines was obtained in a single run with mass spectrometry. The polyamine concentrations were analysed with quadratic discriminant analysis and cross-validated with leave-one-out cross-validation.

**Results:**

Sixty-eight patients with pancreatic cancer, 36 with acute pancreatitis, 18 with chronic pancreatitis and 7 with PLP were recruited, as were 53 controls. The combination of 4 polyamines – acetylputrescine, diacetylspermidine, N^8^-acetylspermidine and diacetylputrescine – distinguished pancreatic cancer and PLP from controls (sensitivity = 94%, specificity = 68% and AUC = 0.88). The combination of diacetylspermidine, N8-acetylspermidine and diacetylspermine distinguished acute pancreatitis from controls (sensitivity = 94%, specificity = 92%, AUC = 0.98). The combination of acetylputrescine, diacetylspermidine and diacetylputrescine distinguished chronic pancreatitis from controls (sensitivity = 98%, specificity = 71%, AUC = 0.93).

**Conclusions:**

Optimally selected urinary polyamine panels discriminate between pancreatic cancer and controls, as well as between acute and chronic pancreatitis and controls.

## Introduction

Pancreatic cancer incidence is rising rapidly, and its projected death rate is among the highest of all cancers.^1–4^ It is difficult to diagnose pancreatic cancer in its early stage because early symptoms are often nonspecific, or absent altogether.^5^ The diagnosis is typically made when the patient has signs of cholestasis, and at that time, 80% of patients will have progressed beyond curative therapy.^6–8^ Due to relatively low prevalence of the disease and expensive, often invasive, follow-up studies, development of strategies for early diagnosis is extremely challenging.^9^ Patients with elevated risk of pancreatic cancer such as those with chronic pancreatitis and premalignant lesions of the pancreas (PLP) could potentially benefit for cancer screening.^10,11^

Polyamines play an important role in cell proliferation, signalling, gene expression, apoptosis and organ development. Spermidine (SPD), spermine (SPM) and putrescine (PUT) are naturally occurring polycationic alkylamines in eukaryotic organs and are essential for cell growth.^12^ Polyamine metabolism plays a key role in the development and growth of pancreatic cancer.^13^ Pancreatic cancer develops after mutations in various oncogenes, which affect polyamine synthesis, leading to increase in intracellular polyamine levels and polyamine spread to tumour tissue, where metabolites are secreted to blood and finally to urine. However, even though some urinary polyamines differ significantly between cancer patients and controls, none have good specificity as a biomarker.^14,15^ The use of multiple polyamines as pattern-recognition and machine-learning algorithms can potentially offer better accuracy than single-polyamine and traditional statistical analyses.

The primary aim of this study was to determine whether pancreatic cancer and PLP can be detected from a urine sample by means of a quantitative analysis of urinary polyamines with liquid chromatography–tandem mass spectrometry (LC-MS/MS).^16–18^ We hypothesized that aggressively growing and invasive pancreatic cancer has a distinct urinary polyamine profile compared to controls without cancer. We analysed 14 polyamines with LC-MS/MS in a single run.^16^ We then identified the polyamine combinations which yield the best ability to detect cancer. The secondary aim was to determine whether acute pancreatitis and chronic pancreatitis can be distinguished from controls and from pancreatic cancer.

## Materials and Methods

Patients were prospectively enrolled at three Finnish hospitals – Seinäjoki Central Hospital, Tampere University Hospital and Kuopio University Hospital – between October 2013 and June 2016 and followed up until April 2017. The inclusion criteria were a new diagnosis of pancreatic cancer or a suspected PLP, acute pancreatitis or chronic pancreatitis. The controls included patients with diagnosed at pelvic prolapse or inguinal hernia. The controls did not have known history of cancer. The controls were enrolled during the same period as the patients with pancreatic cancer, PLP, acute pancreatitis or chronic pancreatitis. The exclusion criteria were a failure in sample preparation or change in diagnosis during follow-up. Pancreatic cancer was diagnosed with CT scan, MRI scan or endoscopic ultrasound. The diagnosis of pancreatic ductal adenocarcinoma was later confirmed with a biopsy and a histological assessment. We used The American Joint Committee on Cancer (AJCC) guidelines for pancreatic cancer staging.^19^ The diagnoses of PLP had been made after tumour biopsy and histology or cytology, and they included adenomas with high-grade dysplasia and mucinous cystic neoplasms (MCN) with high-grade dysplasia but with no sign of pancreatic adenocarcinoma. Intraductal papillary mucinous neoplasms (IPMN) were diagnosed by a radiologist with CT or an MRI scan. Acute pancreatitis was diagnosed by a clinician based on typical abdominal pain, elevated serum amylase and CT or MRI scan with findings suitable for acute pancreatitis. All acute pancreatitis cases were later reviewed from hospital documents and confirmed according to the American College of Gastroenterology Guideline 2013.^20^ Chronic pancreatitis was diagnosed by a clinician based on symptoms (abdominal pain, diarrhoea or weight loss), laboratory tests and CT or MRI scan with findings suitable for chronic pancreatitis. All chronic pancreatitis were later reviewed from hospital documents and confirmed according to the American Pancreatic Association’s diagnostic guidelines in chronic pancreatitis from 2013 with positive CT or MRI imaging, morphology and laboratory testing.^21^ Information on disease characteristics during the follow-up was collected from the patients’ medical records from the time of study enrolment until April 2017. The diagnoses of all enrolled patients who survived were confirmed after a median of 1.9 years’ follow-up (.8–2.9 years). Uncertain diagnoses for patients who died were confirmed from autopsy documents.

We performed first a prospective pilot-study with six patients with pancreatic cancer and 29 controls for sample size estimation to distinguish between pancreatic cancer patients and controls. The power calculation was based on results for individual polyamines with an alpha = 0,05 and power = 0,90.

Written informed consent was acquired from all participants. This study was approved by the Ethical Committee of Tampere University Hospital (code: R10066). The study protocol conforms to the ethical guidelines of the Declaration of Helsinki.

### Determination of Polyamines

Urine sample preparation was performed on the day after the original diagnosis and before cancer surgery. The patients held their bladder 4 hours before producing a 100 ml sample of urine. The controls provided morning urine samples before their hernia operation. The samples were stored at −70 m°C until analysis. The urine samples were collected prospectively without preservatives. All patients continued their normal diets.

The LC-MS/MS analysis was conducted at the University of Eastern Finland (Kuopio, Finland). A detailed description and validation of the LC-MS/MS method used has been published elsewhere.^16^ We analysed the concentration of 14 polyamines including their mono- and di-acetylated forms. The analysed polyamines were diacetylputrescine (DiAcPUT); acetylputrescine (AcPUT); diacetylcadaverine (DiAcCAD); acetylcadaverine (AcCAD); diacetylspermidine (DiAcSPD); 1,3-diaminopropane (DAP); PUT; cadaverine (CAD); N1-acetylspermidine (N1-AcSPD); N8-acetylspermidine (N8-AcSPD); diacetylspermine (DiAcSPM); SPD; N1-acetylspermine (N1-AcSPM) and SPM. Stock solution of each polyamine was prepared by using water as a diluent to get the concentration of 100 mM. We analysed all the 100-mM stock solutions with an nuclear magnetic resonance spectroscopy to ensure the right concentration and purity of the analytes. Afterwards, the stock solutions were further diluted with water to the concentration of 400 μM. Stock solutions were used to make working standard solutions and calibration curve quality control samples. The quality control samples were prepared in human urine. The endogenous polyamine concentrations were measured from the urine of 6 healthy men. Equal amounts of these 6 urine samples were pooled to create a quality control matrix. The matrix was then diluted further with the working standard solution and water to arrive at the working quality control samples. During the sample preparation, solid-phase extraction cartridges were used to remove impurities. We analysed the calibration standard samples before and after each batch and quality control samples between study samples. The acceptance value for intra- and inter-run precision error and for the accuracy for all standards and quality control samples were < 15% and 85%–115%, respectively. The creatinine concentrations of the urine samples were determined enzymatically using the Cobas 6000, C 501- module (Roche diagnostics GmbH, Mannheim, Germany) at Fimlab Laboratories and the urine polyamine concentrations were normalized by the urine creatinine concentration.

### Statistics

The analysis was conducted with MATLAB R2019a (Mathworks Inc, Massachusetts, USA). We used Lilliefors and Jarque–Bera tests to calculate whether urine polyamines follow a normal distribution. None of the polyamines followed normal distribution (*P* = .05) in either of the tests and therefore we employed the Wilcoxon rank sum test to compare medians of each group polyamine concentrations. We applied quadratic discriminant analysis (QDA) with the forward selection method to create classification parameters from the polyamine LC-MS/MS results.^22^ In QDA, the data points are projected to a subspace in which the different classes of the original data are the most distinguishable from each other. To avoid over-fitting, the results were cross-validated using leave-one-out cross-validation (LOOCV). In this method, one by one, each sample polyamine result was first removed from the data pool, and the classification parameters were then created using all the remaining samples as the training set. The single removed sample, acting as the test set, was then classified using these parameters. Optimal polyamine selection was achieved with a forward selection method. A single-polyamine AUC (area under the ROC curve) was determined with an optimal threshold. The AUC for the polyamine profile was arrived at with LOOCV and QDA.

## Results

Due to the prospective pilot-study, the projected sample size needed 46 pancreatic cancer patients in the DiAcSPM group to define differences between pancreatic cancer and control patients and 82 pancreatic cancer patients in the DiAcPUT group, respectively.

Overall, 82 patients with suspected pancreatic cancer were recruited. After a total of 2.9 years of follow-up, 14 patients were excluded. The causes for the exclusions were as follows: diagnoses of cholangiocarcinoma (2 patients), metastasis in the pancreas (1 patient), IPMN (1 patient was moved to the PLP group), pancreatic abscess (1 patient), acute pancreatitis (1 patient was moved to the acute pancreatitis group), a misdiagnosis of pancreatic cancer (1 patient), and sampling failure (7 patients). Finally, 68 patients had a confirmed diagnosis of pancreatic adenocarcinoma and were included in the pancreatic cancer group. Out of the pancreatic cancer patients, 28% had stage IB–IIB and 72% had stage III–IV pancreatic cancer, 74% were inoperable and 26% underwent radical pancreatoduodenectomy or caudal resection (Table 1).

**Table 1.** Clinical Characteristics of Patients and Controls.

Diagnosis	PC	AP	CP	PLP	Controls
N	68	36	18	7	53
Male	36 (53%)	24 (67%)	14 (78%)	4 (57%)	16 (30%)
Age, median (IQR)	71 (64–77)	62 (48–72)	58 (55–65)	63 (58–67)	64 (55–75)
PC stage	—	—	—	—	—
IA	0	—	—	—	—
IB	8	—	—	—	—
IIA	0	—	—	—	—
IIB	11	—	—	—	—
III	21	—	—	—	—
IV	28	—	—	—	—
Non-operable	50	—	—	—	—

PC, pancreatic cancer; AP, acute pancreatitis; CP, chronic pancreatitis; PLP, premalignant lesion of the pancreas; IQR, interquartile range.

The urine polyamine concentrations in a rank-sum test showed significant differences in the concentration of 8 polyamines between pancreatic cancer and control samples (Table 2). DiAcSPD yielded the most significant difference (.50 and .23 μmol/g creatinine, *P* = 6.1 × 10^−10^, respectively). When pancreatic cancer and PLP was analysed as one group, the results were similar. Eight polyamines showed significant differences between acute pancreatitis and the control group. The most significant difference between group medians occurred with DiAcSPM (.55 and .087 μmol/g crea, *P* = 2.1 × 10^−14^, respectively). Between chronic pancreatitis and the control group, 8 polyamines produced significant differences. The most significant difference between the groups appeared with DiAcSPD (.68 and .23 μmol/g crea, *P* = 1.1 × 10^−8^, respectively). Only 2 polyamines, DiAcSPM and SPM, showed a significant difference (*P* = 2.8 × 10^−6^ and .029, respectively) between pancreatic cancer and acute pancreatitis. Patients with acute pancreatitis had higher concentrations of DiAcSPM and SPM in their urine than patients with pancreatic cancer. Furthermore, one polyamine, SPM, yielded a significant (*P* = .018) difference between pancreatic cancer and chronic pancreatitis. Patients with chronic pancreatitis had higher concentrations of SPM in their urine than patients with pancreatic cancer. In the case of 5 polyamines (DiAcCAD, DAP, PUT, CAD and N1-AcSPM), the lowest concentrations were under the lowest calibration point. They were therefore excluded from further analysis.

**Table 2.** The Urine Polyamine Concentrations in Each Pancreatic Disease, μmol/g Creatinine Median (IQR).

	PC		PC + PLP		AP		CP		Controls
	Median	*P*	Median	*P*	Median	*P*	Median	*P*	Median
*DiAcPUT*	.18 (.21)	****	.17 (.21)	****	.13 (.17)	**	.17 (.23)	**	.099 (.072)
*AcPUT*	17.8 (13.5)	****	16.6 (12.0)	****	17.9 (18.2)	****	16.1 (8.01)	***	10.4 (3.57)
*AcCAD*	1.5 (4.4)	****	1.4 (4.3)	****	2.5 (2.9)	****	2.9 (7.7)	***	.54 (1.1)
*DiAcSPD*	.50 (.62)	****	.47 (.50)	****	.59 (.40)	****	.68 (.37)	****	.23 (.084)
*N1-AcSPD*	5.4 (5.1)	****	5.2 (5.2)	****	6.3 (6.0)	****	7.2 (6.1)	****	3.3 (1.7)
*N8-AcSPD*	3.5 (1.9)	****	3.4 (2.0)	****	3.3 (1.9)	**	3.6 (1.7)	***	2.6 (.75)
*DiAcSPM*	.22 (.24)	****	.20 (.24)	****	.55 (.93)	****	.26 (.60)	****	.087 (.049)
*SPD*	.30 (.17)	****	.28 (.17)	****	.36 (.21)	****	.27 (.13)	****	.17 (.081)
*SPM*	.21 (.53)	.47	.20 (.49)	.34	.49 (2.1)	.18	.45 (1.8)	.18	.50 (.97)

PC, pancreatic cancer; AP, acute pancreatitis; CP, chronic pancreatitis; PLP, premalignant lesion of the pancreas; IQR, interquartile range; DiAcPUT, diacetylputrescine; AcPUT, acetylputrescine; AcCAD, acetylcadaverine; DiAcSPD diacetylspermidine, N^1^-AcSPD N^1^-acetylspermidine; N8-AcSPD, N8-acetylspermidine; DiAcSPM, diacetylspermine; SPD, spermidine; SPM, spermine; P, P-value compared to controls. ** P ≤ .01. *** P ≤ .001. **** P ≤ .0001.

**Figure 1.**
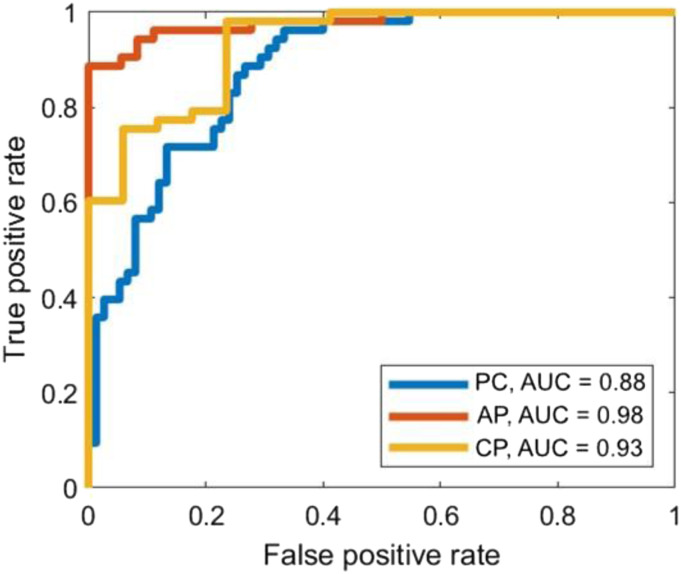
Quadratic discriminant analysis results for selected urine polyamines with an optimal threshold. AUC, area under the ROC curve; PC, pancreatic cancer; AP, acute pancreatitis; CP, chronic pancreatitis; QDA, quadratic discriminant analysis; DiAcPUT, diacetylputrescine; AcPUT, acetylputrescine; AcCAD, acetylcadaverine; DiAcSPD diacetylspermidine; N8-AcSPD, N8-acetylspermidine; DiAcSPM, diacetylspermine; SPM, spermine. PC and PLP are included in the same group and distinguished from controls. Selected polyamines: AcPUT, DiAcSPD, N8-AcSPD and DiAcPUT for PC; DiAcSPD, N8-AcSPD and DiAcSPM for AP and AcPUT, DiAcSPD and DiAcPUT for CP.

In Table 3 and Figure 1, with a forward selection method and quadratic discriminant analysis, 4 selected polyamines, AcPut, DiAcSPD, N8-AcSPD and DiAcPUT, yielded the best AUC when discriminating pancreatic cancer (pancreatic cancer and PLP) from controls (AUC = .88, with a sensitivity of 94% and specificity of 68%). Three selected polyamines, DiAcSPD, N8-AcSPD and DiAcSPM, produced the best AUC for distinguishing acute pancreatitis from controls (AUC = .98, with a sensitivity of 94% and specificity of 92%). But also single polyamine DiAcSPM produced the same AUC when comparing to acute pancreatitis vs the control group. When discriminating chronic pancreatitis from controls DiAcSPD showed the best AUC (AUC = .95), meanwhile in QDA, the best selected combination of polyamines, AcPUT, DiAcSPD and DiAcPUT, showed AUC of .93, respectively.

**Table 3.** Polyamine Panels for Pancreatic Diseases.

	AUC	Sensitivity/specificity%
PC		
QDA (AcPut, DiAcSPD, N8-AcSPD, DiAcPUT)	.88	94/68
AcPut	.74	
DiAcSPD	.82	
N8-AcSPD	.73	
DiAcPUT	.76	
*AP*		
QDA (DiAcSPD, N8-AcSPD, DiAcSPM)	.98	94/92
DiAcSPD	.93	
N8-AcSPD	.68	
DiAcSPM	.98	
*CP*		
QDA (AcPUT, DiAcSPD, DiAcPUT)	.93	98/71
AcPUT	.79	
DiAcSPD	.95	
DiAcPUT	.75	

AUC, area under the ROC curve; PC, pancreatic cancer; AP, acute pancreatitis; CP, chronic pancreatitis; QDA, quadratic discriminant analysis; DiAcPUT, diacetylputrescine; AcPUT, acetylputrescine; AcCAD, acetylcadaverine; DiAcSPD diacetylspermidine; N8-AcSPD, N8-acetylspermidine; DiAcSPM, diacetylspermine; SPM, spermine. PC and PLP are included in the same group and distinguished from controls.

## Discussion

This study shows, for the first time, that pancreatic cancer has distinct urine polyamine profile that can be distinguished from those of healthy controls.

Our results are in line with the literature describing the role of polyamines in pancreatic neoplasia and tumour growth.^13^ Pancreatic cancer progresses slowly from exocrine pancreatic cells from dysplasia to non-invasive precursor lesions and a malignant tumour.^23–25^ Interestingly, the most important gene mutations related with the development of pancreatic cancer, that is, KRAS and MYC, are activators of polyamine metabolism. Mutations in these genes increase the levels of intracellular polyamines that promote tumour growth.^26–28^ Polyamines interact strongly with nucleic acids and chromatin, and are involved in the methylation and acetylation of anionic histones, which interact with RNA and DNA, subsequently affecting protein synthesis, modulating cell growth and proliferation.^29,30^ Chronic inflammation often precedes cancer, and chronic pancreatitis is a known risk factor for pancreatic cancer.^10,11^ Inflammation also affects polyamine metabolism and increases tissue polyamine concentrations.^31,32^ For the purpose of differential diagnosis, we studied urine samples from patients with acute and chronic pancreatitis. Previous studies with an electronic nose showed that there are similarities in the urinary volatile organic compounds (VOCs) of pancreatic cancer and pancreatitis patients.^33–35^ In line with these results, our current study demonstrates that the concentration of urinary polyamines show similar changes in pancreatic cancer and pancreatitis. We could discriminate pancreatic cancer from controls well, but we were only just able to discriminate pancreatic cancer from acute pancreatitis in QDA analysis, with a merely tolerable AUC. Furthermore, in single-polyamine analysis with an optimal threshold, SPM was the only one that yielded a significant difference between chronic pancreatitis and pancreatic cancer. DiAcSPM is supposedly the best-studied polyamine as a novel tumour marker in several cancers.^36–38^ Accordingly, Niemi et al carried out a 14-polyamine analysis, and the best marker for ovarian cancer they found was DiAcSPM. Interestingly, we found that DiAcSPM was significantly higher in patients with acute pancreatitis than in patients with pancreatic cancer. We hypothesize that this is caused by an inflammation that results in more severe disruption of pancreatic cells and higher activation of the immune system than pancreatic adenocarcinoma.

Although altered polyamine levels in body fluids have been reported in many malignancies, the literature on these in pancreatic cancer is limited. Löser et al studied the concentration of 7 polyamines in pancreatic tissue, serum and urine of patients with pancreatic cancer.^39^ Only PUT, CAD and SPD were significantly elevated in pancreatic tissue and serum; all other polyamines, except SPM, were significantly elevated in the urine of cancer patients compared to healthy controls, but the specificity was poor. A signature-based approach was not attempted. Our result of polyamines in the urine of pancreatic cancer was similar: all polyamines were significantly higher in the urine of cancer patients compared to controls, except SPM. In a study of salivary polyamines of pancreatic cancer patients, Asai et al^38^ showed significant differences in SPM, N1-AcSPD and N1-AcSPM concentrations compared to cases and controls. Analogous to our findings from urine, they showed higher salivary N1-AcSPD concentrations in patients with pancreatic cancer compared to controls. The elevation of urinary polyamines is not unique to pancreatic cancer or malignant gastrointestinal diseases.^40^ Even though elevated polyamine levels in blood-, urine- and faecal-based tests have been reported in different cancers, single polyamines have produced low specificity and are therefore not very useful biomarkers for cancer.^41^ We showed for the first time with a very sensitive LC-MS/MS analysis that a combined panel of 4 polyamines yields high sensitivity for pancreatic cancer and the panel has a good AUC. Additionally, a panel of three selected polyamines differentiates acute and chronic pancreatitis from controls.

The study had several important limitations. Firstly, we had to exclude 5 polyamines, DiAcCAD, DAP, PUT, CAD and N1-AcSPM, from the analysis due to concentration results under the lowest calibration point in the LC-MS/MS analysis. Secondly, we had to exclude 14 patients due to missing urine sample or a diagnosis other than pancreatic cancer either in a histological examination or during follow-up. The strengths of our study were the long follow-ups that enabled us to rule out misdiagnoses made at study entry with reasonable confidence. With the small proportion of patients with resectable pancreatic cancer, we had a representative group of patients with PLP, the majority of which had high-grade dysplasia. The validation of the results with LOOCV increases the likelihood that the results are reproducible. To eliminate urine concentration bias, the urine polyamine concentrations were normalized by the urine creatinine concentration.

Although polyamines have a significant role in tumour growth and they have been proposed as a therapeutic target in pancreatic cancer, the literature on their role in the early diagnosis of pancreatic cancer is scarce. By utilizing advanced mathematical methods, we demonstrated that the combination of selected polyamines can distinguish even early-stage pancreatic malignancies with a good AUC. We acknowledge that since the incidence of pancreatic cancer even in a selected population is low, a diagnostic test needs to have better specificity to deliver clinical value. In the future, the analysis could be combined with symptom reports and general blood markers, such as fasting glucose, and with well-known cancer markers such as CA19-9. Since acquiring CEA and CA19-9 were not part of the study protocol, they were only available for a few patients in our sample, preventing any meaningful analysis.

As a final note, polyamines are also VOCs,^42^ which are known to have a strong odour. PUT and CAD are foul-smelling polyamines, which are best-characterized as components that smell of death.^43^ Dogs have been shown to detect various cancers from urine,^44–46^ which in the concept level shows that the smell of cancer exists. Field asymmetric ion mobility spectrometry (FAIMS) can detect pancreatic cancer from urinary gaseous headspace.^33,34^ However, it is not known which urinary VOCs are specific to pancreatic cancer.^35^ Polyamines are released in large quantities in putrefaction and their foul odour may assist canines in detection of carcasses and cancer. We hypothesize polyamines could be the explanatory compounds.

## Conclusion

In summary, we demonstrated the detection of pancreatic cancer from urine samples by means of a quantitative analysis of urinary polyamines with LC-MS/MS and QDA analysis. We showed that a combination of polyamines performs better than individual polyamines in the discrimination of pancreatic cancer from controls. These findings implicate the possibility to develop a non-invasive test for the early diagnosis of pancreatic cancer.

## Appendix A

### Abbreviations

AcCAD: acetylcadaverine
AcPUT: acetylputrescine
AP: acute pancreatitis
AUC: area under the ROC curve
CA19-9: carbohydrate antigen
CAD: cadaverine
CP: chronic pancreatitis
DAP: 1,3-diaminopropane
DiAcCAD: diacetylcadaverine
DiAcPUT: diacetylputrescine
DiAcSPD: diacetylspermidine
FAIMS: field asymmetric ion mobility spectrometry
IPMN: Intraductal papillary mucinous neoplasms
LC-MS/MS: liquid chromatography–tandem mass spectrometry
LOOCV: leave-one-out cross-validation
MCN: mucinous cystic neoplasms
N1-AcSPD: N1-acetylspermidine
N1-AcSPM: N1-acetylspermine
N8-AcSPD: N8-acetylspermidine
PC: pancreatic cancer
PLP: premalignant lesions of the pancreas
PUT: putrescine
QDA: quadratic discriminant analysis
SPD: spermidine
SPM: spermine
VOCs: volatile organic compounds.
